# Supplementary material for: Crowdsourcing temporal transcriptomic coronavirus host infection data: Resources, guide, and novel insights
Source: Biol Methods Protoc. 2023 Nov 14;8(1):bpad033. doi: 10.1093/biomethods/bpad033 (PMC10723038; doi:10.1093/biomethods/bpad033)
Supplement: bpad033_Supplementary_Data [file bpad033_supplementary_data.zip › BSCE Supplemental Text.pdf]

BaseSpace™ Correlation Engine for Mining Genomic Data

Bringing Big Data and Biology Together

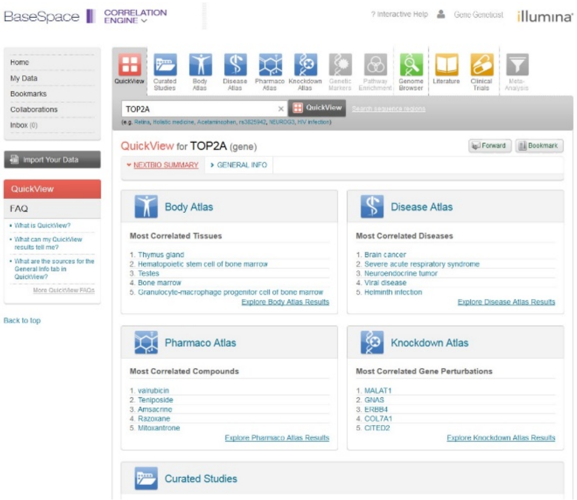

Fig 1. BaseSpace Correlation Engine reveals data driven connections between genes, diseases, compounds, tissues, pathways and literature. Novel correlations and associations are quickly identified for a given query. Adaptive learning processes take advantage of weekly updated content to organically compute ranked association scores for tissues, diseases, compounds and genetic perturbations from public and proprietary data.

Illumina's BaseSpace™ Correlation Engine (BSCE, formerly known as NextBio™ Research) provides life science researchers with unprecedented access to vast numbers of high quality curated public whole genomic studies and insightful scientific tools (Body Atlas, Disease Atlas, Pharmaco Atlas, Knockdown Atlas, Genetic Markers, Meta-analysis). A simple intuitive graphical interface (Fig 1) built to take advantage of continuously expanding content enables researchers to easily and quickly identify novel correlations. This powerful software as a service (SaaS) application allows users to seamlessly share data across organizations and across multiple geographic locations enabling more impactful collaborations and partnerships.

How It Works

Public data are evaluated both programmatically and by stringent scientific review before they are uploaded into BSCE. Over 80,000 studies have been evaluated to arrive at the more than 22,000 that have been included to date. A study must be of a supported species, have sufficient replicates, use a supported whole genome technology (e.g., not PCR), have recognizable features if array based, have a sensible study design (i.e., treated vs. untreated, disease vs. normal, etc.), use unique samples (not a re-analysis) and pass data quality assurance. For NGS studies, raw data is required, and a minimal read depth and RNA quality standards are imposed. For arrays, raw data is preferred, and processed data must be untransformed and normalized in a way comparable to a per-chip median normalization process (e.g., RMA, MASS5) with no evident batch effects. Authors are contacted whenever annotations are unclear or discrepancies are found. New studies are added on a continuous basis[1].

The primary analysis result is referred to as a **bioset**, which is a list of elements (genes, probes, proteins, compounds, single-nucleotide variants [SNVs], sequence regions, etc.), ranked or unranked, that corresponds to a given treatment or condition versus a baseline or reference condition in an experiment. For a gene expression experiment, biosets are derived for all relevant experimental factors within a study and consist of gene signature lists with associated fold change values and statistical information such as p-values and q-values. Only statistically significant results will be reported for a given experiment. For example, an RNA-Seq dataset is subject to statistical thresholds set at a minimum 1.2 absolute fold change and a maximum adjusted p-value (q-value) of 0.05. Finally, each bioset is associated with an array of ontology-based biomedical concepts which determine how query results are organized in the system (see below).

The statistics associated with gene elements in a bioset is used to determine their ranks and directionality[2]. In brief, statistics selected by the user (e.g., fold change) are converted to non-

parametric ranks, while fold change direction gives those ranks a positive or negative sign (Fig 2A). A mapping logic is defined to derive corresponding genes when studies measure chromosomal regions or SNPs[2]. Subsequent enrichment analysis is performed on the derived set of mapped genes and associated ranks (Fig 2B). In cases where a user imports a custom gene list without ranks or direction, a simplified enrichment analysis is applied[2].

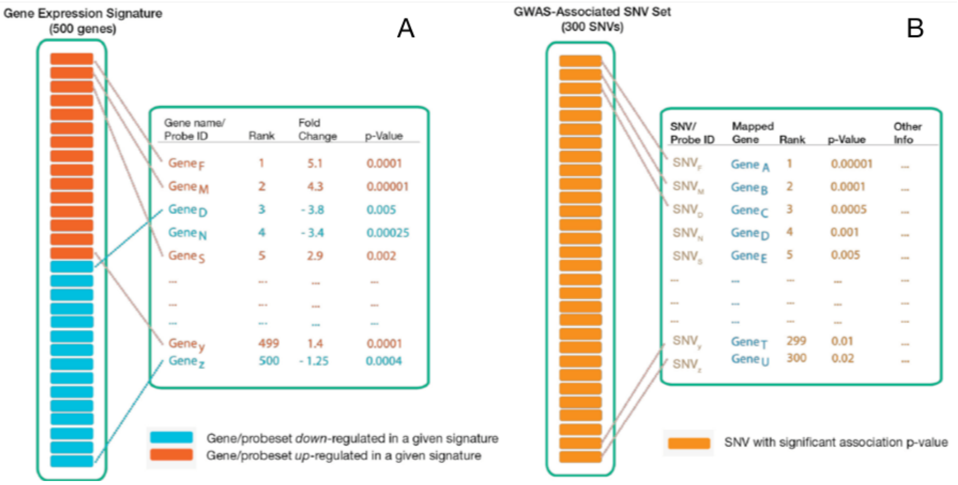

Fig 2. Bioset Ranking and Directionality *Examples of ranking in different bioset types. A) ranks based on fold change and directionality* (fold change direction). *B) GWAS association bioset with a set of mapped genes, used in the enrichment analysis. Ranks for SNPs and mapped genes are derived from the corresponding p-value.*

Illumina has developed a specialized gene set enrichment algorithm that forms the basis to perform a variety of analyses. The general design of the algorithm (**Running Fisher**) aims to compare a query signature with a target signature, which is analogous to the Gene Set Enrichment Analysis (GSEA) method[3,4]. Our Running Fisher algorithm dynamically detects the most significant enrichment signal in a ranked signature set, allowing the signature set to contain relatively more comprehensive collections of genes at a preselected statistical cutoff. The Running Fisher algorithm differs from GSEA in the assessment of the statistical significance, where p-values are computed by a Fisher's exact test rather than by permutations (Fig 3A). Also, unlike GSEA, the Running Fisher can evaluate gene sets with both up and down regulated genes. Overall, the advantage of this approach is the flexibility of being able to compute correlation scores for data of different types, sizes, and filter thresholds. The algorithm involves identifying subset pairs defined by available directionality in the data. The Running Fisher algorithm is applied to each subset pair (Up *vs.* Up, Down *vs.* Down, Up *vs.* Down, Down *vs.* Up; Fig 3B). The genes in the query subset are scanned top to bottom in the rank order to identify each rank with a gene matching a member in the target signature. If the subset is unranked, all the genes in the subset are retrieved at the first scan. At the end of the scan, the best p-value is retained, and the negative log of the p-value is a score for the subset pair. Next, the Running Fisher algorithm is performed with the previous query signature as the target and the previous target signature as the query

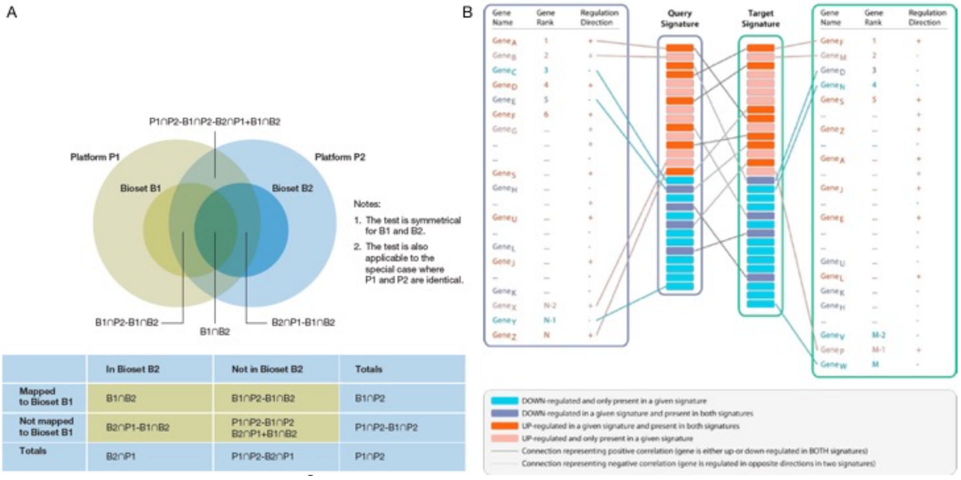

directional, the direction-specific subset (b1+ or b1- in place of B1; b2+ or b2- in place of B2) is used. The scan of the bioset dynamically cuts off the top K rows of the gene set for evaluation at rank K, where K is a dynamic number from 1 to N in the query bioset or 1 to M in the target.

signature, and the average of the two best scores is taken to measure the magnitude of similarity between the two subsets.

The overall correlation score is the sum of directional subset scores, and the sign of the sum determines whether the two signatures are positively or negatively correlated (Fig 4A). If one gene set

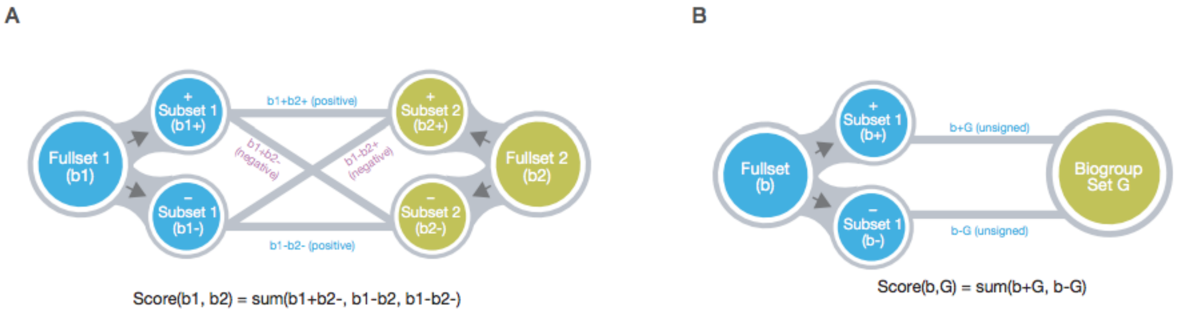

Fig 4: Rank-Based Directional Enrichment. Enrichment scores are computed for the directional subsets, followed by a summation over all the subset scores. A) Bioset vs. Bioset: the subsets are formed for both b1 and b2, and subset-subset enrichment scores are computed. B) Bioset vs. unranked gene set: the subsets are formed for the bioset, and subset-biogroup enrichment scores are computed.

is ranked and directional while the other is not, enrichment is computed for each directional subset (b+, b-) in the ranked, directional bioset. The overall enrichment score is the sum of the subset scores (Fig 4B). In the application, scores are presented in top-level results normalized to a linear 0 to 100 scale with 100 representing the highest score. The magnitude of this overall enrichment score is also used to rank the query to every other bioset in the system (including user data in their private domain) and rendered top to bottom in the Curated Studies app[3,5].

Ontology-Driven Meta-Analysis

Every bioset entered into the system is specifically 'tagged' with ontology-based biomedical terms for associating and ranking top data-driven diseases, compound treatments, and genetic perturbations.

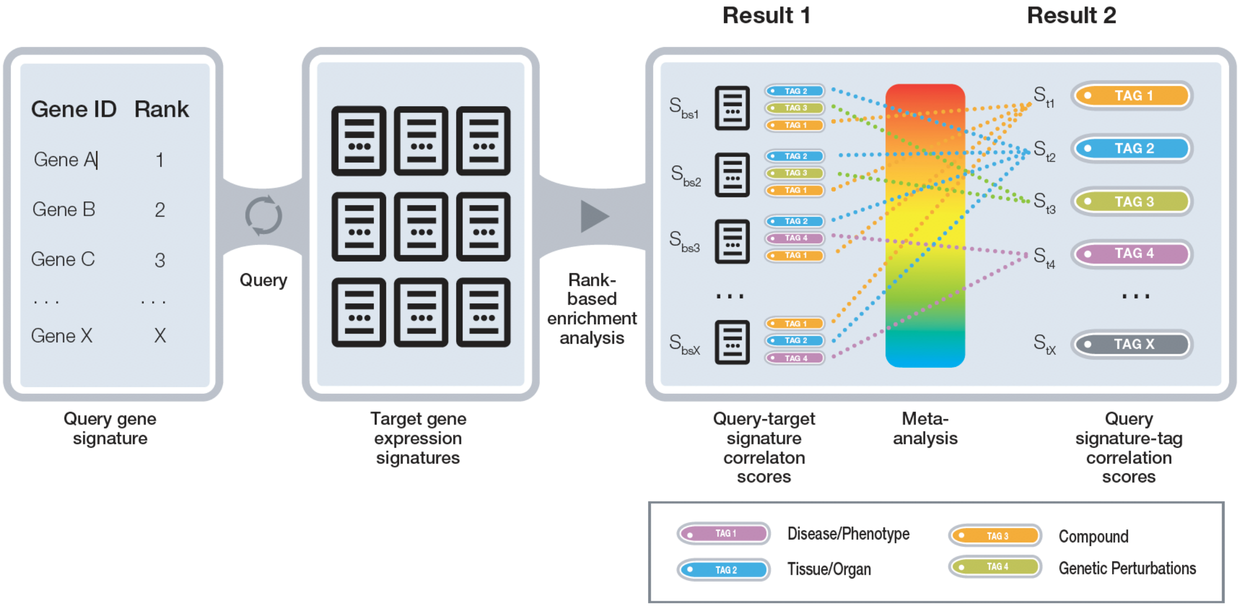

Fig. 5. Data Correlation and Meta-analysis. Pairwise gene bioset correlation scores (computed using rank-based enrichment statistics) are computed, follow by meta-analysis of individual score-tag pairs to compute overall tag scores.

The bioset-bioset scores are used to compute scores against these ontology terms, which in turn are

used to rank results in the Disease, Pharmaco, and Knockdown Atlases. For a gene or region query, the inverse of the normalized rank of the gene or gene region in the target biosets is used (see below). When a gene or gene signature is queried, the first result is the rank ordered list of biosets scored from best down to a 10^{-6} cutoff. These biosets will then be categorized based on their tags and used to compute scores for each of the associated ontology terms (Fig 5). In the Disease Atlas, for example, this will produce a rank ordered list of disease categories based on a score computed from the tagged biosets.

A number of factors feed into the concept score (Equation 1). The *NormalizedBiosetCount* is the sum of the ratio of scores to the best score (or in the case of a gene query, the sum of the ratio of inverse ranks to the best rank). This is divided by the *BackgroundCount* which is the total number of biosets tagged with a given concept, both scored and below the scoring cutoff. This important factor balances against undue significance being given to over-represented concepts in the system. The concept score is further reduced by the *AverageWeightedRank,* which is a measure of how well the query signature scores against the concept data compared to all signatures in the system. Queries that perform better against the concept versus all signatures will be have an *AverageWeightedRank* closer to 1 and a better overall score.

Equation 1. $$Score_{Concept\ or\ Gene} = \frac{NormalizedBiosetCount}{BackgroundCount\ x\ AverageWeightedRank}$$

Start today!

The public data available in **BaseSpace Correlation Engine** is just the starting point for discovery. Users can upload their own data and query it against itself and the public data. Enterprise account holders can share their results within their private domain and add their results to the meta-analysis applications to generate unique correlations. Private data is inaccessible across domains and results are kept safe and private in an ISO27001, SOC1, SOC2, SOC3, PCI DSS certified environment.

To purchase, start a free trial and learn more go to **www.illumina.com/basespacecorrelationengine.**
For additional contact information, please see **https://www.illumina.com/company/contact-us.html**.
Special academic pricing available.

References

1. BaseSpace Correlation Engine Support page – https://support.illumina.com/sequencing/sequencing_software/basespace-correlation-engine/documentation.html
2. Ranking of Genes, SNVs, and Sequence Regions – https://support.illumina.com/content/dam/illumina-marketing/documents/products/technotes/technote-ranking-snvs.pdf
3. Ontology-Based Meta-Analysis of Global Collections of High-Throughput Public Data Kupershmidt I, Su QJ, Grewal A, Sundaresh S, Halperin I, Flynn J, Shekar M, Wang H, Park J, Cui W, Wall GD, Wisotzkey R, Alag S, Akhtari S, Ronaghi M. PLoS One. 2010 Sep 29;5(9). pii: e13066. doi: 10.1371/journal.pone.0013066. PMID: 20927376
4. Gene set enrichment analysis: a knowledge-based approach for interpreting genome-wide expression profiles. Subramanian A, Tamayo P, Mootha VK, Mukherjee S, Ebert BL, Gillette MA, Paulovich A, Pomeroy SL, Golub TR, Lander ES, Mesirov JP. Proc Natl Acad Sci U S A. 2005 Oct 25;102(43). PMID: 16199517
5. Data Correlation Details: Enrichment Analysis – https://support.illumina.com/content/dam/illumina-marketing/documents/products/technotes/technote-data-correlation-enrichment.pdf

For Research Use Only. Not for use in diagnostic procedures.

© 2018 Illumina Inc. All rights reserved. QB 6281
