# Supplementary figures and images for: Crowdsourcing temporal transcriptomic coronavirus host infection data: Resources, guide, and novel insights
Source: Biol Methods Protoc. 2023 Nov 14;8(1):bpad033. doi: 10.1093/biomethods/bpad033 (PMC10723038; doi:10.1093/biomethods/bpad033)

Supplemental Figure 1

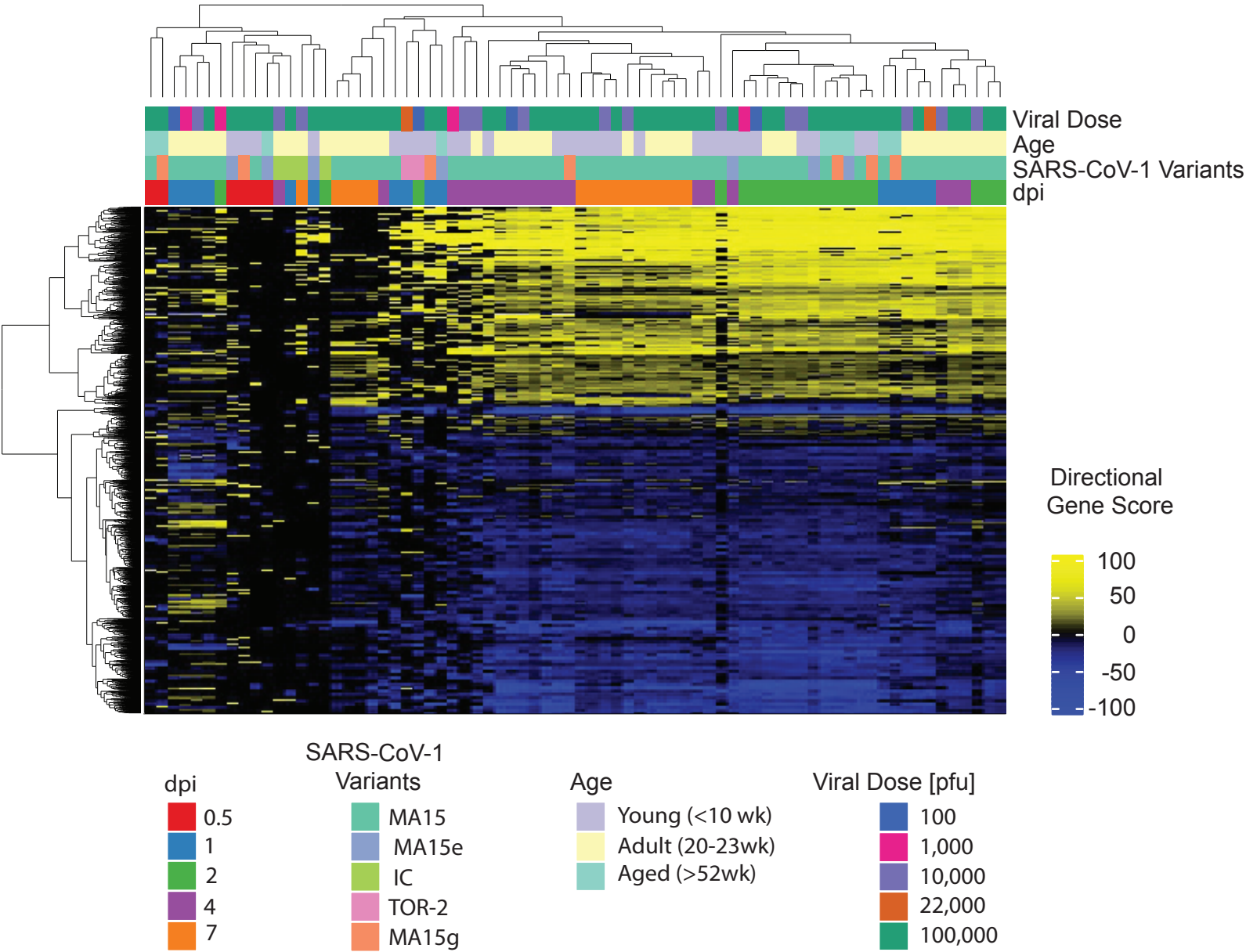

Supplement: bpad033_Supplementary_Data [file bpad033_supplementary_data.zip › Supplemental Figure 1 - Two-way Agglomerative Hierarchical Clustering Heatmap of 74 SARS-CoV-1 Biosets.pdf]

Supplemental Figure 2

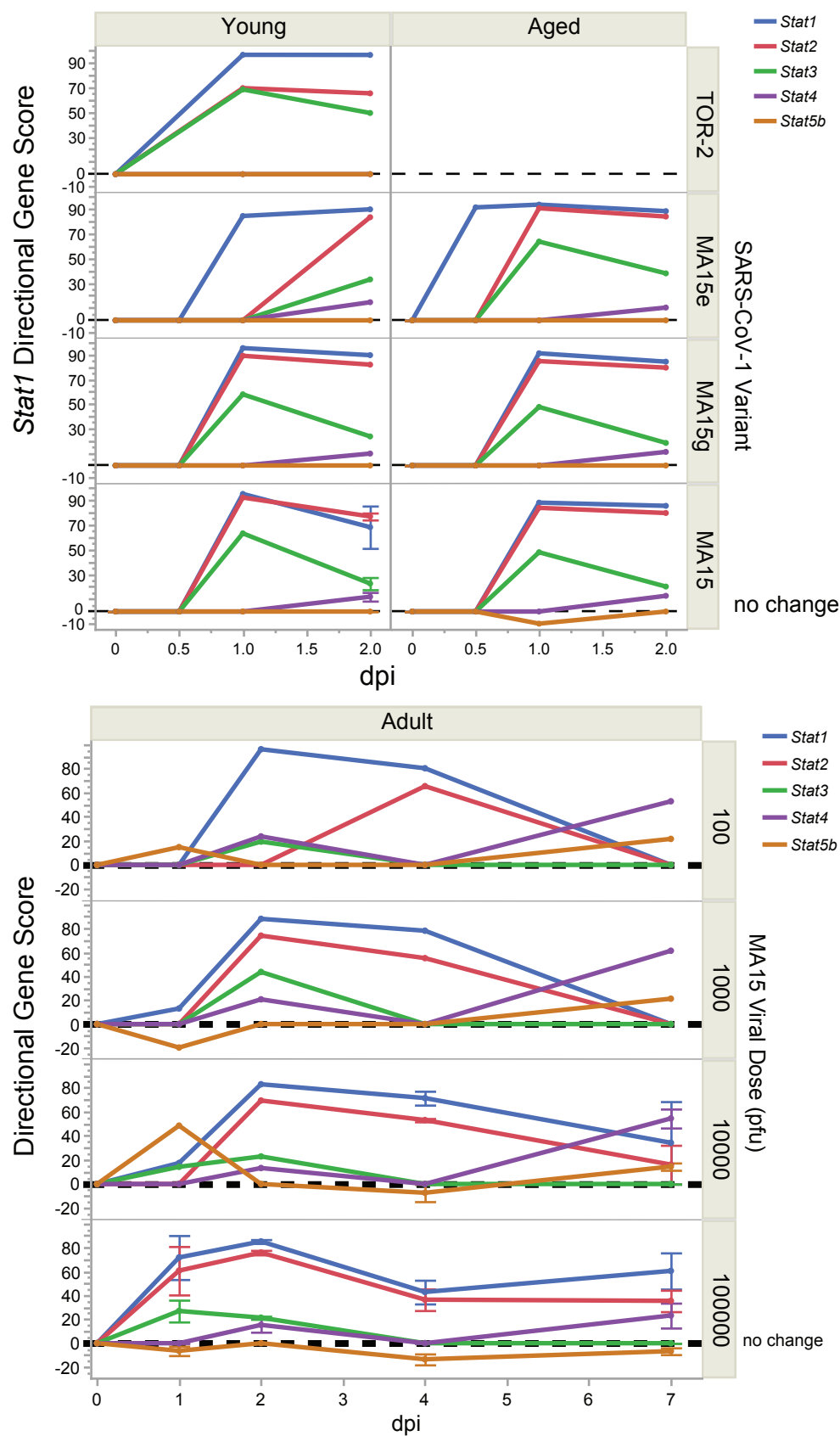

Supplement: bpad033_Supplementary_Data [file bpad033_supplementary_data.zip › Supplemental Figure 2 - Analysis of Stat genes by host age viral variant and viral dose.pdf]

Supplemental Figure 3

A

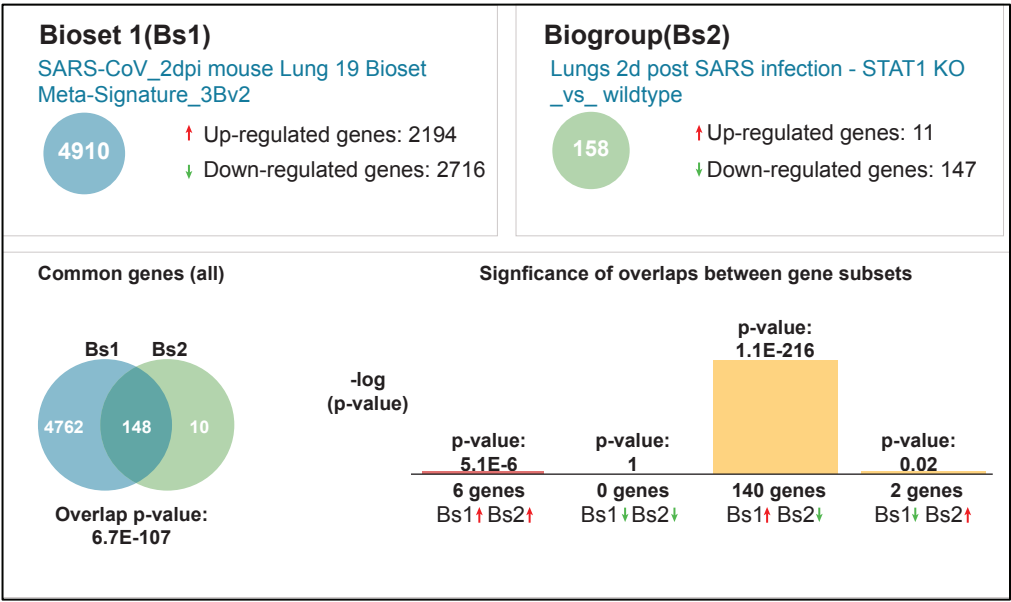

B

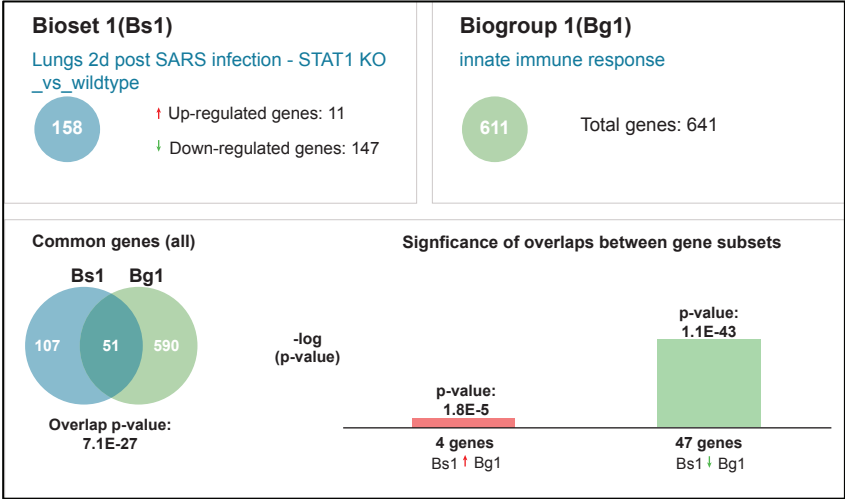

C

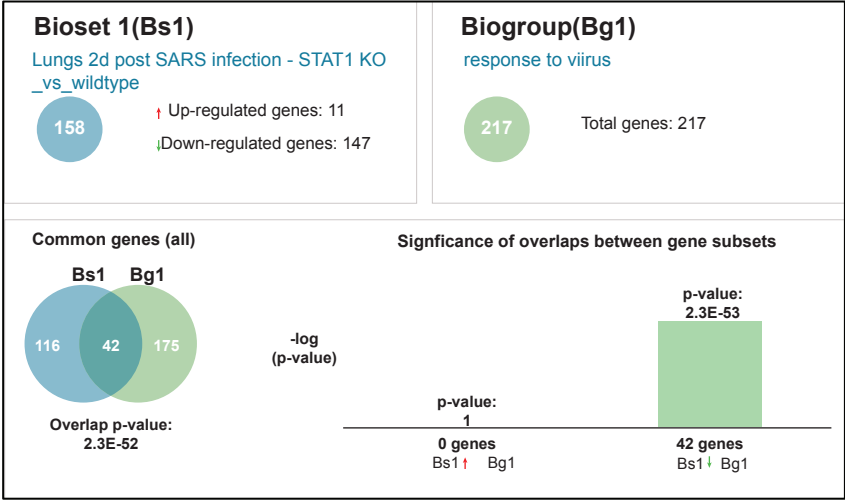

Supplement: bpad033_Supplementary_Data [file bpad033_supplementary_data.zip › Supplemental Figure 3 - Correlation of Stat1 Knockout Transcriptional Data to SARS-COV-1 Meta-signatures.pdf]

Supplemental Figure 4

down-regulated at 1,2 dpi

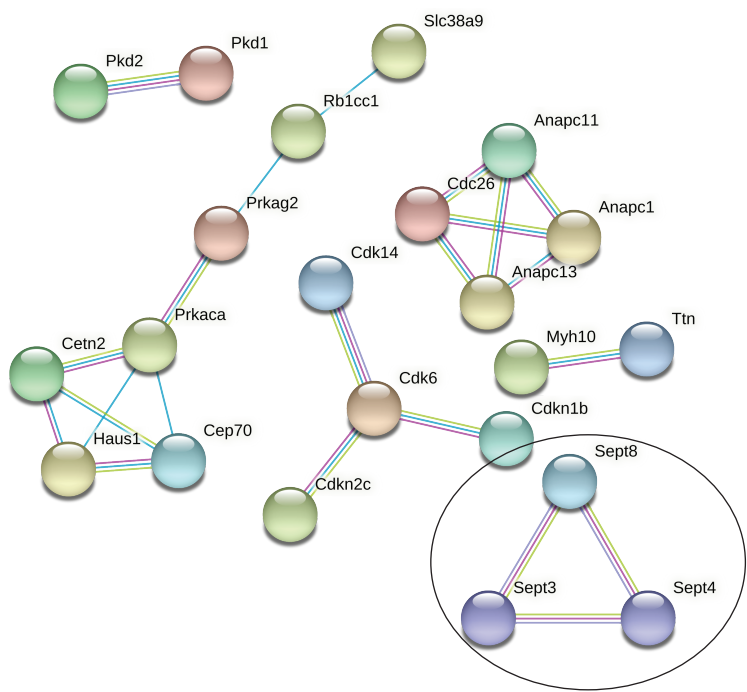

down-regulated at 4,7 dpi

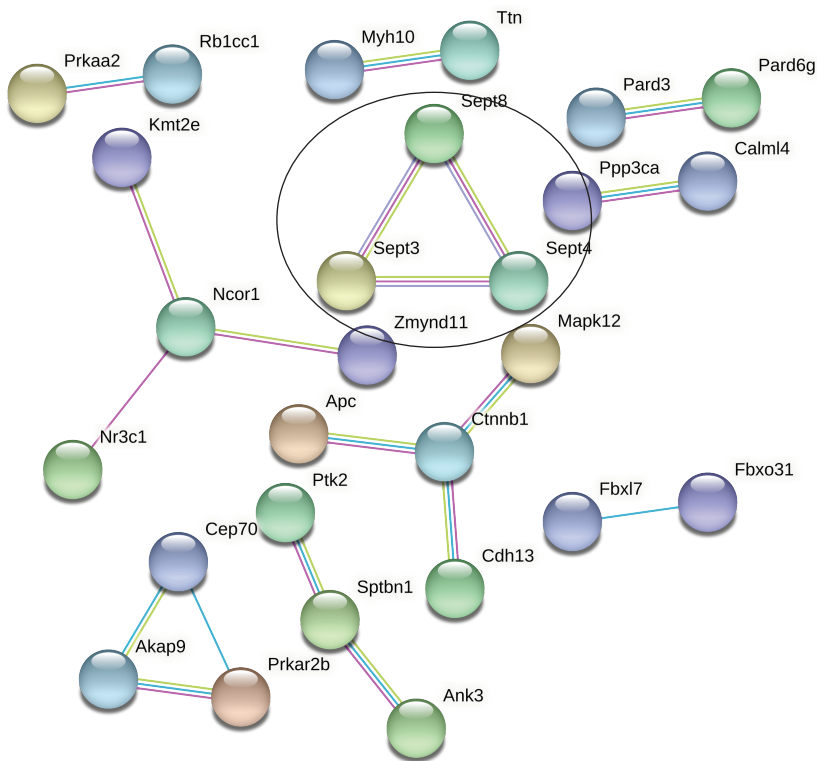

Supplement: bpad033_Supplementary_Data [file bpad033_supplementary_data.zip › Supplemental Figure 4 - Down-regulated Cell Cycle Genes in Early and Late Infection.pdf]
